# Dynamic Changes in Dietary Guideline Adherence and Its Association with All-Cause Mortality among Middle-Aged Chinese: A Longitudinal Study from the China Health and Nutrition Survey

**DOI:** 10.3390/nu15061401

**Published:** 2023-03-14

**Authors:** Xiao Zhang, Xiaona Na, Yanfang Wang, Shufa Du, Ai Zhao, Wannian Liang

**Affiliations:** 1 Vanke School of Public Health, Tsinghua University, Beijing 100084, China; xiz186@pitt.edu (X.Z.);; 2 Institute for Healthy China, Tsinghua University, Beijing 100084, China; 3 Peking University Clinical Research Institute, Beijing 100083, China; 4 Gillings School of Global Public Health, University of North Carolina at Chapel Hill, Chapel Hill, NC 27599, USA

**Keywords:** dietary guideline, dietary quality, mortality, China Health and Nutrition Survey, longitudinal study

## Abstract

The traditional approach to evaluating dietary quality is based on the achievement of the recommended intakes for each food group, which may overlook the achievement of correct relative proportions between food groups. We propose a “Dietary Non-Adherence Score (DNAS)” to assess the degree of similarity between subjects’ diets and those recommended in the Chinese Dietary Guidelines (CDG). Furthermore, it is important to incorporate the time-dependent nature of dietary quality into mortality prediction. This study investigated the association between long-term changes in adherence to the CDG and all-cause mortality. This study included 4533 participants aged 30–60 from the China Health and Nutrition Survey study with a median follow-up of 6.9 years. Intakes from 10 food groups were collected in 5 survey rounds from 2004 to 2015. We calculated the Euclidean distance between the intake of each food and the CDG-recommended intake, and then summed all the food groups as DNAS. Mortality was assessed in 2015. Latent class trajectory modeling was used to identify three classes of participants with distinct longitudinal trajectories of DNAS during the follow-up period. The Cox proportional hazard model was used to assess the risk of all-cause mortality in the three classes of people. Risk factors for death and confounders for diets were sequentially adjusted in the models. There were 187 deaths overall. Participants in the first class identified had consistently low and decreasing DNAS levels (coefficient = −0.020) over their lifetime, compared with a hazard ratio (HR) of 4.4 (95% confidence interval [CI]: 1.5, 12.7) for participants with consistently high and increasing DNAS levels (coefficient = 0.008). Those with moderate DNAS had an HR of 3.0 (95% CI: 1.1, 8.4). In summary, we find that people with consistently high adherence to CDG-recommended dietary patterns had a significantly lower mortality risk. DNAS is a promising method to assess diet quality.

## 1. Introduction

Dietary factors have been related to healthy longevity and are among the top five attributable risk factors for death worldwide [1]. The Chinese Dietary Guidelines (CDG) is the official dietary guideline that has been designed to encourage healthy, habitual food choices, decrease chronic disease risk and improve public health [2]. The CDG recommends a diet high in grains, vegetables, and fruits, with moderate consumption of meat, poultry, eggs, and dairy products [2]. Higher adherence to the CDG was associated with reduced risks of all-cause mortality [3], and mortality from cancer [4] or cardiovascular disease [5]. Traditional a priori approaches measure the extent to which individuals adhere to dietary recommendations and assess the population’s overall dietary quality, for example, the Alternate Healthy Eating Index (AHEI) [6], Dietary Quality Score [7], the Mediterranean diet score [8], and the Chinese Diet Balance Index [9]. The healthier foods a person eats and the fewer unhealthy foods he or she eats, the higher the total score obtained. However, two respondents with the same total score may have different scores on individual foods and subsequently have different health effects. For example, someone who scores 0 on dairy and 10 on red meat (i.e., no dairy, no red meat) will have a lower total calorie intake than someone who scores 10 on dairy and 0 on red meat (i.e., the maximum amount of dairy and red meat). To address this problem, we propose the use of a “Dietary Non-Adherence Score (DNAS)” as a vector-based approach, benchmarked against the Euclidean distance. This score measures the degree of dissimilarity between a subject’s dietary pattern and the recommended pattern from the CDG. Unlike traditional dietary quality scores, DNAS evaluates the correct proportion between food groups holistically as opposed to the adequacy of any individual food. We proposed that the DNAS could predict mortality and thus serve as a tool for evaluating diet quality.

In addition, the dietary pattern may change over the course of a lifetime. First, as people age, their calorie intake decreases and their food intake becomes less varied [10] due to loss of appetite, decreased chewing ability [11], and multimorbidity [12]. Second, from the 2000s to the 2020s, due to economic growth, the Chinese diet gradually diversified [13] and gradually increased the intake of vegetables, fruits, snacks, dairy, and animal products, and steadily decreased the intakes of cereals and tubers [14]. The above changes in diet structure led to an increased prevalence of cardiometabolic diseases and other chronic diseases in the Chinese population [15,16,17]. No studies have examined the association between changes in dietary dynamics during midlife and mortality in the Chinese population.

Because of the critical role diet plays in health and the need to incorporate the time-dependent nature of dietary patterns into mortality prediction, this study aimed to investigate long-term changes in non-compliance with the CDG and all-cause mortality, using data from the China Health and Nutrition Survey (CHNS).

## 2. Materials and Methods

**Figure A1:**
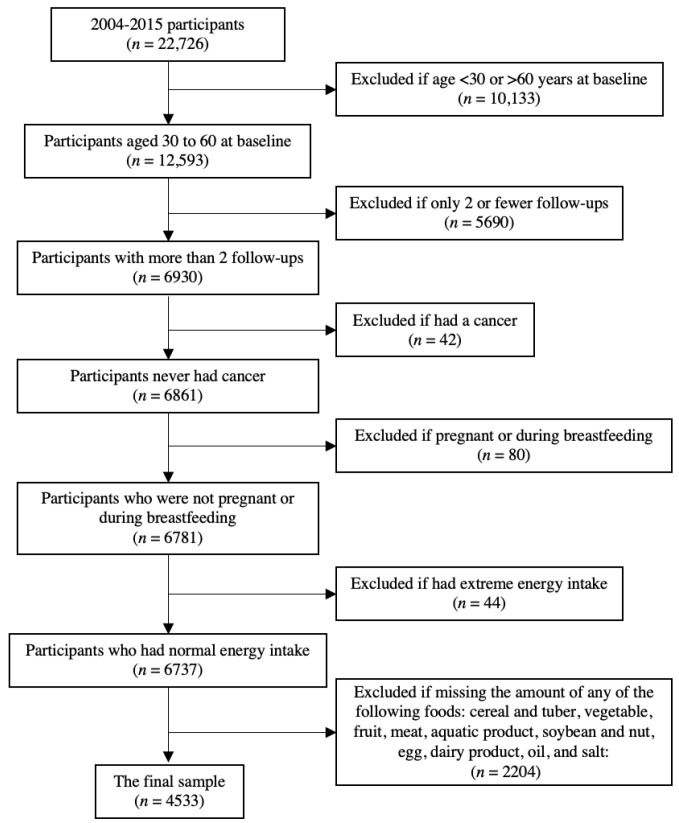
Flowchart of sample selection.

The China Health and Nutrition Survey (CHNS) is a nationwide prospective cohort study. The initial recruitment of participants was conducted in 1989, and follow-ups were conducted within a 2–3-year interval. Participants were recruited from nine provinces and three autonomous cities. The detailed description can be found elsewhere [18]. In this study, we used the data from the CHNS collected in 2004, 2006, 2009, 2011, and 2015. Since the dietary data for 2015 are not yet available, only mortality data were included from that interval. We excluded elderly people (>60 years at baseline) because they tend to consume fewer calories [10] and may suffer from multimorbidity [19] and thus may have significantly different dietary patterns to younger people. We also excluded people aged <30 years at baseline because we want to capture dietary change along with aging. Furthermore, we excluded people who met the following criteria: during pregnancy or lactation during investigation, only participated in ≤2 follow-ups, had cancers, had extreme energy intake (<500 or >8000 kcal/day), missing the amount of food groups, or missing key covariates (i.e., individual income, smoking status, chronic disease history, taking medicine, and physical activity), resulted in a total sample of 4533 in the final analysis (Figure A1).

The CHNS was approved by institutional review boards at the University of North Carolina (Chapel Hill, NC, USA) and the National Institute of Nutrition and Food Safety (Chinese Center for Disease Control and Prevention). Informed consent was given to all participants before participation. The current study was further approved by the Institution Review Board of Tsinghua University (project identification 20210072).

Dietary data were collected by trained interviewers over three consecutive days within a week at individual and household levels [20,21,22]. The three consecutive days were selected randomly from Monday to Sunday. For individual dietary intake, all the foods consumed (meals and snacks) by participants over the previous 24 h were reported. Types, quantities of all food consumed, and dining places were recorded by the interviewers with the help of food models and pictures. Household food and condiment consumption were calculated by recording the changes in inventory from the beginning to the end of a three-day survey, including all purchased, homemade, and processed food. In the present study, the amount of the following foods was measured by the 24 h dietary recall: cereals and tubers, vegetables, fruits, meat, aquatic products (e.g., fish and shellfish), soybean and nuts, eggs, and dairy products; and measured at household levels: edible oil, salt and other condiments. Energy intake from both food and condiment at each meal was calculated by the China Food Composition.

The Euclidean distance [23] or some monotonic transformation of it, such as the mean squared error, is often used as a loss function in statistics, and is employed to evaluate the amount of “similarity” between two objects, each of which is decomposed into a fixed number of components, and dissimilarity is then modeled as a metric in the resulting feature space [24]. The Euclidean distance has been used in medicine to investigate patient similarity, such that to identify patients who agreed the most with each patient, to enable a better prediction of certain health outcomes [25,26]. For example, David et al. proposed an algorithm for anomaly detection and characterization on the basis of the Euclidean distance between the medical laboratory data [26]. With the selected neighbors around him, the index patient could be segmented into one of the seven disease groups with a higher accuracy. In another study, for the early screening and assessment of suicidal risks, researchers used the sum of absolute distances for each predictor to retrieve a cohort of similar patients so that the researchers could determine the most potential risk level for a new patient [27].

**Figure A2:**
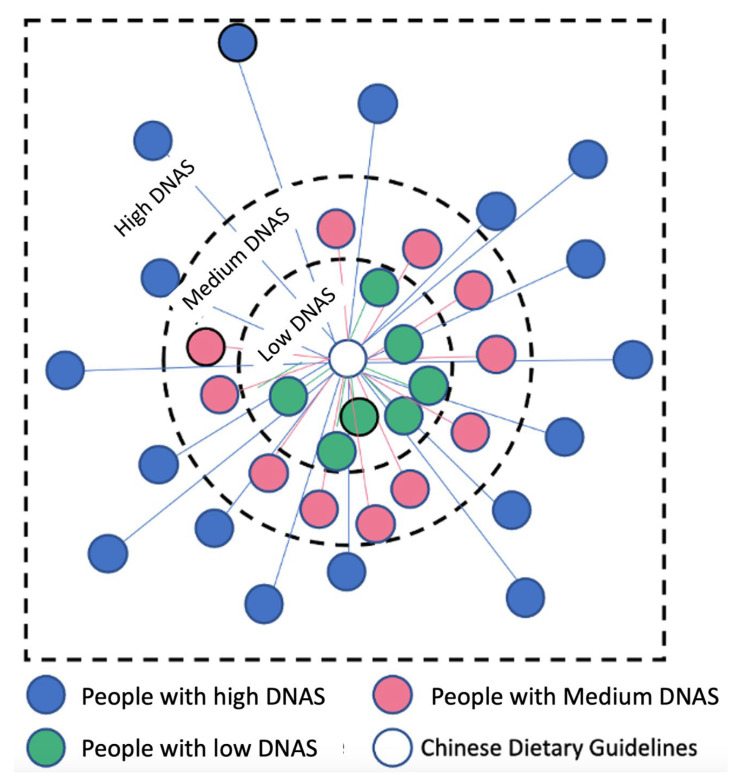
The schematic of the classification of the population according to Diet Adherence Score category. The central circle represents the Chinese Dietary Guideline (CDG). The surrounding dots represent people in the sampled population. The distance from each individual to the CDG is the distance in a 10-dimensional space from each individual’s intake of 10 food groups to the median of the intake range specified in the CDG.

We found the DNAS by adding up the distance between the actual and recommended intake of each food group using the Euclidean method. The Euclidean distance was calculated as $d_{n}\left(x,y\right)=\sqrt{\sum_{i=1}^{n}{\left(x_{i}-y_{i}\right)}^{2}}$ [23], where $x_{i}$ is the actual intake of each food group for an individual and $y_{i}$ is the median of the recommended intake range according to the CDG. The recommended range in the CDG and the values used in our analysis can be found in Table A4. As illustrated in Figure A2, the central point indicates individuals who follow the recommended dietary pattern exactly, while the points on the periphery represent those who deviate from it to varying degrees, with the furthest points indicating the greatest deviation. The DNAS is the sum of vectors in a ten-dimensional space, as it takes into account ten different food groups.

The primary outcome of the present study is all-cause mortality. For each participant in the CHNS, the household register system would continuously update their status, either alive or deceased, and the year and month of death. The year of follow-up was calculated from enrollment to the date of passed away or loss of follow-up of the participant during 2004–2015.

In order to model DNAS as a function of age, we used latent class trajectory modelling (LCTM) to identify subgroups of participants with distinct trajectories over the study period. Detailed mathematical equations were described by previous studies [28,29]. We used maximum likelihood approaches to fit the model with the “hlme” function [30] from “lcmm” library in the R software environment (version 1.9.3). The call of “hlme” fits (i) a standard liner mixed model in which the dependent variable DNAS is explained by age, and (ii) a 2-class linear mixed model similar to (i) but with the effect of age different among classes. Age was modelled with the random effect and in the liner pattern because our interest lies in the variation among the sampled population rather than the specific effects of each level, and that the polynomial form of age was not significant. We determined the optimal number of classes (i.e., 3) based on the lowest Bayesian information criteria.

The characteristics of all eligible participants are summarized as the mean and standard deviation (SD) for continuous variables and frequencies and percentages for categorical variables. The variations in the characteristics across the classes were analyzed using analysis of variance for continuous variables and chi-square test for categorical variables.

Cox proportional hazard regression was applied to test the association between DNAS trajectories and the risk of all-cause mortality. We built Cox proportional hazard models by adding confounders measured at baseline in a sequential manner, based on their level of association with mortality and diet: (i) age, sex, and region of residence; (ii) chronic disease history (diabetes, hypertension, and cardiovascular disease), using hypotensive or hypoglycemic medicine, current smoker, physical activity, total energy intake, and body mass index; and (iii) current alcohol drinker, individual annual income (yuan), and educational level.

The hazard of mortality and its relationship with continuous, baseline DNAS was investigated using a restricted cubic spline Cox regression, with adjustments made for any confounding factors, as the linear trend test met the significance level. The regression model with three knots was selected because it has the largest coefficient of determination (R^2^) among all candidate models. All statistical analyses were performed using R Statistical Software (version 4.1.1, R Development Core Team, Vienna, Austria). *p*-value < 0.05 (two tailed) was considered statistically significant.

## 3. Results

In total, 4533 eligible participants were included in the analysis, 50.5% women and 49.5% men. The median follow-up time was 6.9 years; 187 cases of death occurred during the follow-up years. The DNAS varies between 0.8 and 8.6.

### 3.1. Baseline Characteristics in Three Classes

**Figure 1:**
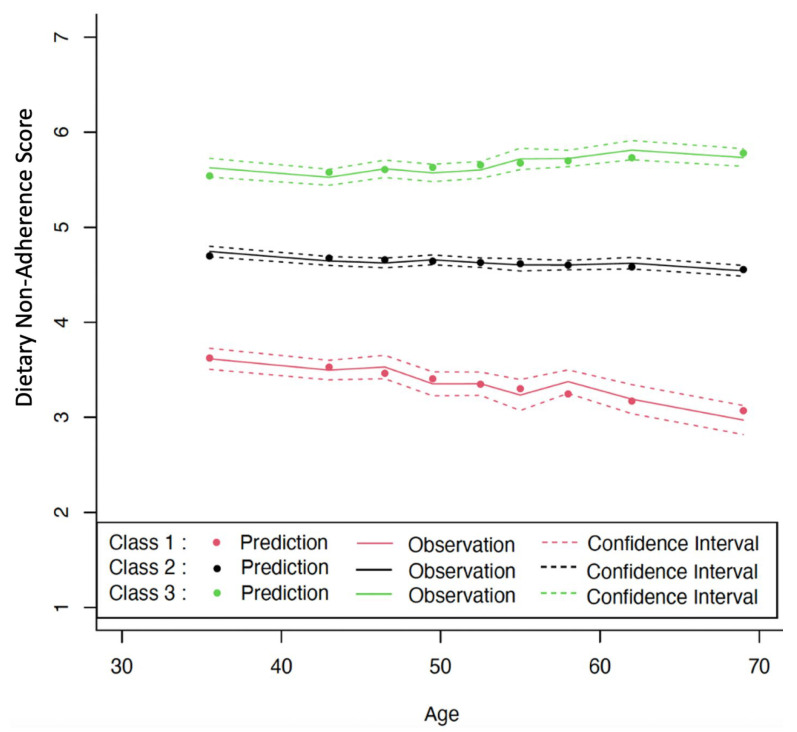
Levels and changes in Dietary Non-Adherence Score (DNAS) over chronological age. Estimated from latent class trajectory modeling. Class 1 = low DNAS, class 2 = medium DNAS, and class 3 = high DNAS.

Figure 1 displays the graph for the final model chosen by LCTM with class 1 shown on the bottom, class 2 in the middle, and class 3 at the top. Individuals whose change profiles are most closely aligned with class 1 tended to have the lowest levels of DNAS that decreased over time (coefficient = −0.020, *p* < 0.001), indicating high compliance with the guideline and increasing compliance over their lifetime. Class 3’s DNAS consistently increased over time (coefficient = 0.008, *p* < 0.001), showing a decline in adherence to the guideline as time progressed. Class 2 barely changed over time.

Table 1 shows the baseline characteristics in the three classes. Classes 1, 2, and 3 had mean DNAS values of 3.3, 4.8, and 5.9, respectively. The mortality rates (95% CI) were 11.8 (4.5, 31.5), 28.8 (23.5, 35.3), and 44.5 (30.6, 64.9) per 1000 person-years, respectively. There were no significant differences in age, BMI, or the frequency of chronic disease or drinking alcohol across classes. However, as the classes increased, physical activity level, mortality rate, and the frequency of smoker increased (*p* < 0.05), while individual income level and frequency of taking medicine decreased (*p* < 0.05).

The median consumption of different food groups among participants of different classes is presented in Table A1. As class level rose, there was a significant increase in cereal and tuber consumption, as well as salt intake (*p* < 0.05). However, there was a significant decrease in the consumption of meat, eggs, soybeans, nuts, and edible oil (*p* < 0.05).

### 3.2. Association of DNAS with Mortality

As class level increased, the hazard ratios (HR) of all-cause mortality also increased (Table 2). In comparison to class 1, the unadjusted HRs (95%CI) for classes 2 and 3 were 4.6 (1.7, 12.5) and 8.3 (3.0, 23.1), respectively. Adjusting for demographic factors such as age, sex, and region slightly decreased the estimates to 4.0 (1.5, 10.8) and 6.5 (2.3, 18.1). Further adjustment for risk factors such as chronic disease, medicine use, smoking, physical activity, energy intake, and BMI resulted in HRs (95%CI) of 3.7 (1.4, 10.2) and 5.9 (2.1, 16.6). Finally, when adjusted for other risk factors such as alcohol consumption, income, and education, the HRs (95%CI) were 3.0 (1.1, 8.4) and 4.4 (1.5, 12.7) for classes 2 and 3, respectively.

**Figure 2:**
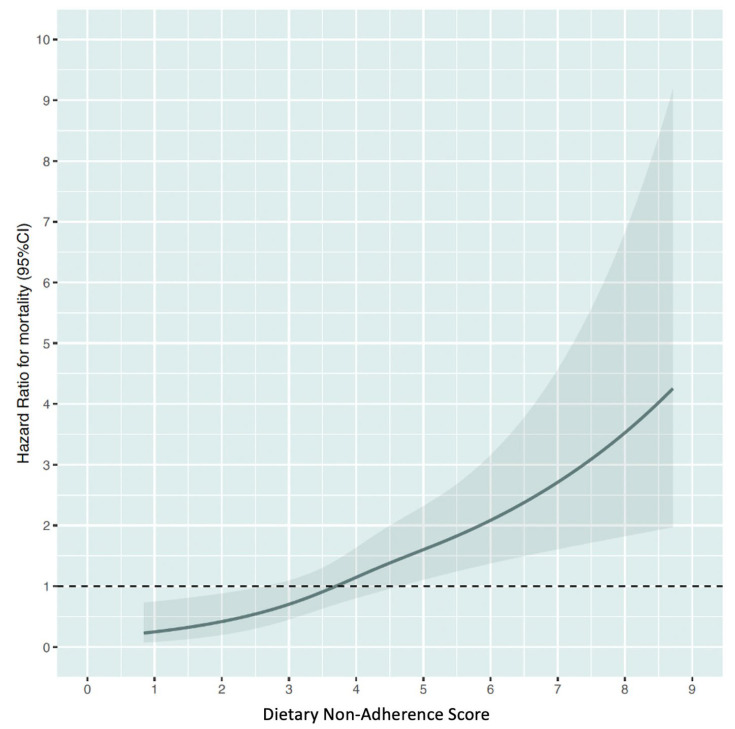
Relationship between Dietary Non-Adherence Score and the risk of all-cause mortality. Estimated from restricted cubic spline Cox regression modelling.

The restricted cubic spline curve revealed a positive and consistent relationship between DNAS and mortality risk, as shown in Figure 2. Those with DNAS lower than 3.6 can lower their risk by following a healthy diet, while those with DNAS 3.6 or higher may see an increase in risk due to poor dietary habits.

## 4. Discussion

The DNAS is a measure we developed to evaluate the extent to which the proportion of various food components in an individual’s diet deviates from the recommended ratios established by the CDG. As demonstrated in Chinese adults, an individual’s DNAS score can change throughout their life. Both the initial DNAS level and the trajectory of DNAS over time are effective indicators of risk for all-cause mortality. Individuals with high DNAS scores that continue to increase over time have a 4-fold higher risk of death compared to those with low DNAS scores that decrease consistently over time.

Chinese adherence to dietary guidelines remains suboptimal. Among all participants, 11% deviated significantly from the optimal food intake ratio and this gap continued to widen with age, 76% deviated to some degree and remained unchanged, and only 13% had the appropriate intake ratio, which improved with age (Figure 1). The fifth national survey on nutrition (2010–2013) found that the average daily food intake for Chinese individuals was 337 g of cereals and tubers, 269 g of vegetables, 41 g of fruits, 90 g of red meat and poultry, 24 g of aquatic products, 24 g of eggs, 25 g of dairy products, 42 g of oil, and 11 g of salt, which is approximately 90% less dairy products, 80% less fruits, 30% less vegetables, and 20% less aquatic products compared to the guideline. The persistent gap between recommendations and implementation is likely the result of a combination of cultural influences, societal norms, family influences, personal food preferences, food availability and accessibility, declining food preparation skills, food marketing practices, time pressures, and economic realities [31,32,33].

DNAS is a unique approach that combines the advantages of both investigator-driven and data-driven methods. DNAS is distinct from other dietary scores such as the AHEI and the Mediterranean diet score because it does not have predetermined score ranges or values for each component of the dietary score, eliminating subjective interpretation by researchers in terms of guidelines. Furthermore, DNAS ensures overall balance of the diet by considering the correlation of different dietary components, which is a characteristic of posterior methods such as principal component analysis. Additionally, people with middle-range scores often have diverse nutritional compositions and dietary patterns, but traditional dietary scores fail to reveal these distinctions. DNAS, however, can accurately quantify these differences.

**Figure A3:**
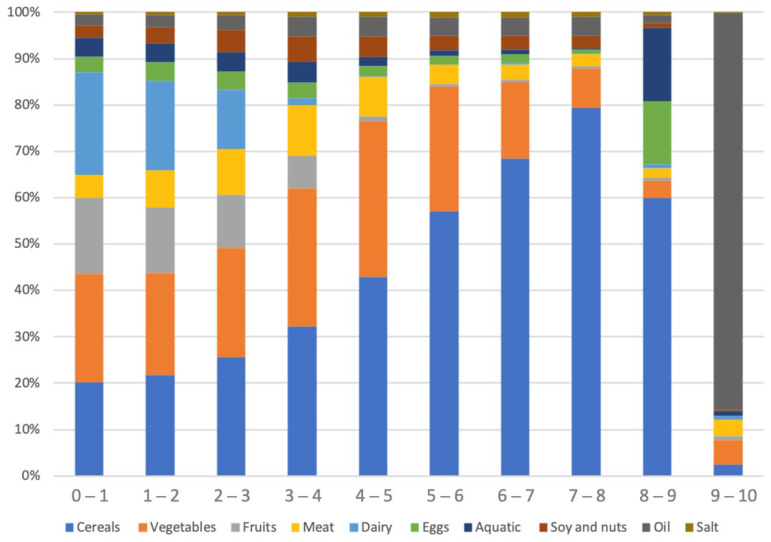
Weight of each food as a percentage of the total weight of all food groups within the range of DNAS.

The traditional diet score typically informs us of the quantity of specific food groups, with a higher score indicating a greater presence of nutritious food or a lower presence of unhealthy food. In contrast, our diet score prioritizes the overall balance of all food groups rather than the quantity of individual food groups. Still, the group that adhered more closely to the guideline had a diet that included more meat, eggs, soybeans, and nuts, while the group that had low adherence to the guideline had a diet that was higher in cereals, tubers, and salt (Figure A3). An adequate intake of high-quality protein may have positive effects on health [34]. Additionally, it has been established that refined grains have a lower protective effect in preventing chronic diseases [35]. As the level of DNAS increased, the percentage of certain food groups such as cereals, vegetables, meat, soy and nuts, and salt initially rose, but eventually dropped (Figure A3).

The effectiveness of a diet score is determined by both its ability to accurately reflect dietary preferences and its ability to predict disease [36]. DNAS meets these criteria, as our study showed that deviation from recommended food intake ratios can increase the risk of death. Previous studies have indicated that a higher adherence to dietary recommendations, both Chinese and American, is associated with a lower risk of death. Research has shown that higher Chinese Food Pagoda scores are associated with lower all-cause mortality in about 140,000 Chinese adults when extreme quartiles are compared (HR [95%CI]: 0.67 [0.60, 0.75] in men, 0.87 [0.80, 0.95] in women) [3]. An analysis among 8 cohorts (about 514,000 subjects) found that a 2-point increase in adherence to a Mediterranean diet is associated with a 9% decrease in mortality risks (95% CI: 0.89, 0.94) [37]. The AHEI is a widely used measure of dietary quality (Table A2) that has been linked to the risk of cardiovascular disease, diabetes, and other chronic diseases [38,39], and has been found to be associated with not only mortality from all-cause [40], but also cardiovascular disease [40,41] and cancer [42]. While the impact of DNAS is reduced after accounting for the AHEI, it still holds statistical significance (Table A3). Thus, DNAS can be used as a dependable method to assess adherence to the guideline.

Our study is the first to demonstrate that the relative proportion of food groups in the Chinese diet does not vary significantly with aging. On the other hand, previous research has indicated that the absolute amount of Chinese food consumed does change over time. In this manner, our study does provide insights into the overall picture of Chinese dietary pattern. Cross-sectional studies have found that older people are less likely than younger adults to consume red meat, whole milk and other fatty foods, and are more likely to consume fruits and vegetables [43,44]. Longitudinal data, including one study based on the CHNS, supports that this represents actual age differences and not just a cohort effect [45,46,47]. The differences with age may be due to the significantly lower digestive capacity of the elderly [10] and their greater susceptibility to mineral and vitamin deficiencies [48]. Our findings imply that for advocating increased adherence to dietary guidelines, residents probably need to focus not only on the adequacy of food, but also on the relative amount of food.

We acknowledge several limitations in this analysis. Firstly, DNAS, which reflects the absolute distance, does not indicate over- or underconsumption of specific food groups. However, it has been found to predict the risk of death and offers a unique perspective in the field. Secondly, adherence to the guideline in 2022 does not necessarily indicate adherence to previous editions, as the guideline has been updated multiple times in the past. However, the dietary guidelines have remained largely unchanged since 2007, with only small adjustments to recommended amounts of certain food groups. Additionally, the CHNS survey and the release of guidelines do not align in terms of timing, so the guidelines cannot be used for the CHNS survey conducted in the same year. Furthermore, using a consistent diet measurement over time enables us to make valid comparisons between different points in time and minimize errors. Thirdly, the number of deaths is small because our sample was restricted to middle-aged individuals. This is because older people tend to have distinct dietary pattern as a result of having multiple chronic health conditions. Fourthly, we recognize the possibility of residual confounding, such as the influence of urbanicity, which may be indicated by dietary patterns, social norms, and environmental factors.

To sum up, using the DNAS to track dietary habits over time can accurately predict the risk of death in a group of Chinese people between the ages of 30 and 60. The DNAS offers additional insights into an individual’s dietary habits and is therefore a promising method for assessing dietary quality.

## Figures and Tables

**Table 1:** Characteristics of participants across classes identified by latent class trajectory modeling (N = 4533).

	All	Class 1 (Low DNAS)	Class 2 (Medium DNAS)	Class 3 (High DNAS)	*p*-Value
	N = 4533	N = 378	N = 3522	N = 633	
DNAS	4.8 (1.0)	3.3 (1.0)	4.8 (0.8)	5.9 (0.9)	<0.001
Age at entry, year	43.3 (8.3)	43.8 (8.6)	43.0 (8.2)	44.7 (8.3)	0.17
Physical activity (MET, min/week)	3099 (2837)	1518 (1652)	3158 (2856)	3714 (2959)	<0.001
BMI (kg/m^2^)	23.3 (3.2)	23.6 (3.1)	23.4 (3.2)	23.1 (3.3)	0.65
Energy intake (cal/day)	2224 (503)	2121 (400)	2236 (501)	2221 (557)	<0.001
Individual annual income (yuan)	10,909 (18,056)	18,393 (16,906)	10,933 (19,194)	6307 (7680)	<0.001
Chronic diseases	32.0%	35.2%	32.4%	30.2%	0.25
Took medicine	19.2%	24.1%	19.2%	16.4%	0.01
Current smoker	43.1%	29.6%	43.1%	48.7%	<0.001
Current drinker	52.5%	52.4%	52.6%	52.1%	0.98
Mortality rate (1000 person-years) (95% CI)	29.6 (24.8–35.3)	11.8 (4.5–31.5)	28.8 (23.5–35.3)	44.5 (30.6–64.9)	<0.001

DNAS, Dietary Non-Adherence Score; MET, metabolic equivalent of task; BMI, body mass index; CI, confidence interval. Mean (standard deviation) or percentage are shown for continuous and categorical variables, respectively. The range for DNAS is between 0.8 and 8.6. Chronic disease included in this table: diabetes, hypertension, and cardiovascular disease; medicine: hypotensive or hypoglycemic medicine.

**Table 2:** Hazard Ratios (95% Confidence Interval) of all-cause mortality for classes identified by latent class trajectory model (N = 4533).

	Class 1	Class 2	Class 3
	(Low DNAS)	(Medium DNAS)	(High DNAS)
Model 1	1.0 (ref)	4.6 (1.7, 12.5)	8.3 (3.0, 23.1)
Model 2 (+ age, sex, region)	1.0 (ref)	4.0 (1.5, 10.8)	6.5 (2.3, 18.1)
Model 3 (+ disease, medicine, smoker, physical activity, energy intake, BMI)	1.0 (ref)	3.7 (1.4, 10.2)	5.9 (2.1, 16.6)
Model 4 (+ drinker, income, education)	1.0 (ref)	3.0 (1.1, 8.4)	4.4 (1.5, 12.7)

Model 1: unadjusted model. Model 2: adjusted for age at entry, sex, region of residence. Model 3: additionally adjusted for chronic disease history, taking medicine, current smoker, physical activity, total energy intake, body mass index. Model 4: additionally adjusted for current alcohol drinker, individual annual income, educational level. DNAS, diet adherence score; BMI, body mass index. Mortality rate (95% CI) (per 1000 person-years): class 1 = 11.8 (4.5, 31.5), class 2 = 28.8 (23.5, 35.3), and class 3 = 44.5 (30.6, 64.9).

## Data Availability

China Health and Nutrition Survey (CNHS) is available at https://www.cpc.unc.edu/projects/china/data/datasets/index.html (accessed on 1 October 2022). Analytic code used in this study will be made available upon request.

## Appendix A

**Table A1:** Food intake (gram/day) at baseline by class identified by latent class trajectory modeling.

	Class 1 (Low DNAS)	Class 2 (Medium DNAS)	Class 3 (High DNAS)
	N = 378	N = 3522	N = 633
Cereals and tubers	341 (269–433)	446 (350–558)	608 (439–792)
Vegetables	307 (217–383)	340 (240–480)	267 (166–412)
Fruits	83 (0–183)	0 (0–0)	0 (0–0)
Meat	97 (60–160)	66 (17–133)	0 (0–47)
Aquatic products	33 (0–83)	0 (0–50)	0 (0–0)
Eggs	40 (16–67)	16 (0–40)	0 (0–20)
Dairy products	83 (0–200)	0 (0–0)	0 (0–0)
Soybeans and nuts	42 (0–88)	33 (0–80)	0 (0–50)
Salt	8 (5–12)	9 (6–14)	10 (7–13)
Oil	44 (27–69)	42 (27–63)	33 (19–53)

DNAS, Dietary Non-Adherence Score. Median (25–75th percentile) is shown. All comparisons are statistically significant at 0.05 level.

**Table A2:** Scoring method of the AHEI (2010).

Population	Component	Criteria for Minimum Score (0)	Criteria for Maximum Score	Maximum Score Value
American	Vegetables, servings/d	0	≥5	10
	Fruit, servings/d	0	≥4	10
	Whole grains, g/d	0		10
	Women		75	
	Men		90	
	Sugar-sweetened beverages and fruit juice, servings/d	≥1	0	10
	Nuts and legumes, servings/d	0	≥1	10
	Red/processed meat, servings/d	≥1.5	0	10
	trans Fat, % of energy	≥4	≤0.5	10
	Long-chain (n-3) fats (EPA + DHA), mg/d	0	250	10
	PUFA, % of energy	≤2	≥10	10
	Sodium, mg/d	Highest decile	Lowest decile	10
	Alcohol, drinks/d			10
	Women	≥2.5	0.5–1.5	
	Men	≥3.5	0.5–2.0	
	Total			110

**Table A3:** Hazard Ratios (95% Confidence Interval) of all-cause mortality for classes identified by latent class trajectory model.

	Class 1	Class 2	Class 3
	(Low DNAS)	(Medium DNAS)	(High DNAS)
	N = 378	N = 3522	N = 633
Model 5 (all confounders + AHEI)	1.0 (ref)	1.2 (1.1, 9.0)	3.1 (1.9, 5.1)

Adjusted for age, gender, region, chronic disease history, medicine, current smoker, physical activity, energy intake, body mass index, current alcohol drinker, individual annual income, educational level, the AHEI, the Hazard Ratio (95% Confidence Interval) for the AHEI is 0.98 (0.96, 0.99). DNAS, diet adherence score; AHEI: Alternative Healthy Eating Index.

**Table A4:** Recommended food intake range in the CDG, the median value within this range, and the proportion of this food group among all food groups.

Food Groups	Ranges (gram)	Taking Values (gram)	Percentage
Cereals and tubers	Cereals 200–300, tubers 50–100	325	19.9
Vegetables	300–500	400	24.5
Fruits	200–350	275	16.8
Meat	120–200 (including aquatic products)	60	3.7
Aquatic products	≥2 times per week	60	3.7
Soybeans and nuts	25–35	30	3.1
Eggs	1 per day	50	24.5
Dairy products	300–500	400	1.8
Edible oil	25–30	27.5	1.7
Salt	<5	5	0.3
